# Prevalence and patterns of soft tissue metastasis: detection with true whole-body F-18 FDG PET/CT

**DOI:** 10.1186/1471-2342-7-8

**Published:** 2007-12-12

**Authors:** Nghi C Nguyen, Bassem T Chaar, Medhat M Osman

**Affiliations:** 1 Division of Nuclear Medicine, Department of Radiology, Saint Louis University Hospital, St. Louis, USA; 2 Division of Hematology and Oncology, Department of Internal Medicine, Saint Louis University Hospital, St. Louis, USA

## Abstract

**Background:**

The aim of this retrospective study was to report the prevalence and patterns of soft tissue (ST) metastasis detected with true whole-body (TWB) F-18 FDG PET/CT acquired from the top of the skull through the bottom of the feet and to compare such findings to that of the typically acquired skull-base to upper-thigh, thus limited whole-body (LWB) field of view (FOV).

**Methods:**

TWB FDG-PET/CT scans were performed in 500 consecutive cancer patients. Suspected ST metastasis was verified by correlation with surgical pathology, other imaging modalities, or clinical follow-up.

**Results:**

Nine out of 500 patients (1.8 %) had ST metastasis with a prevalence of 4/41 (9.8%) for melanoma, 2/60 (3.3%) for lung carcinoma, 2/88 (2.3%) for lymphoma and 1/13 (77%) for esophageal cancer. Those nine patients had a total of 41 ST lesions: 22 lesions within and 19 outside of LWB FOV. Of those 41 lesions, 19 (46%) were subcutaneous and 22 (54%) were muscular lesions. The presence of ST metastasis neither changed the staging nor the treatment in any of these patients. However, the ST lesions provided a biopsy site in 4 of the 9 patients (44%). Seven out of nine studied patients died of their disease within 1–22 months after ST metastasis was diagnosed.

**Conclusion:**

The detection of ST metastasis may have prognostic implications, provide more accessible biopsy sites and help avoid invasive procedures. A LWB scanning may underestimate the true extent of ST metastasis since a significant percentage of ST metastasis (46%) occurred outside the typical LWB FOV.

## Background

Distant metastasis to ST, defined as metastasis to skeletal muscle and subcutaneous tissues, are rarely reported in the literature. Autopsy series have reported ST metastasis in 0.75%-9% of patients who died of metastatic carcinoma [1-3]. The detection of ST metastasis may affect staging and prognosis. Accurate tumor staging encompassing the entire body is important. There is a growing body of literature regarding the added value of F-18 FDG PET/CT in cancer patient management [4]. In oncology, whole body PET/CT is typically performed from the skull base to the pelvic floor [5,6] because most FDG avid lesions are expected within this field of view. This FOV correlates with that of diagnostic CT scans when separate scans of the neck, chest, abdomen and pelvis are performed. If the primary tumor or the suspected metastatic site is outside the LWB, the FOV is then extended to cover this site, thus, allowing proper diagnosis, staging and restaging. The LWB FOV may underestimate the true extent of ST metastasis by missing lesions outside this FOV. To our knowledge, there have been no studies systematically evaluating ST metastasis by F-18 FDG PET/CT. The aim of this study was to report the prevalence and patterns of ST metastasis detected with True Whole-Body (TWB) F-18 FDG PET/CT, from the top of the skull through the bottom of the feet, and to compare such findings to that of the LWB FOV. Further, the implications of ST metastasis on prognosis and patient management were evaluated.

## Methods

### Patients

A total of 500 consecutive patients referred for clinical evaluation of known malignancy and who had undergone a PET/CT scan between September 2004 and February 2005 were retrospectively evaluated.

### Inclusion and exclusion criteria

Criteria for inclusion were the presence of pathologically proven malignancy and the development of metastatic ST lesions in the skeletal muscles and/or subcutaneous tissues confirmed by histopathology, clinical diagnosis or other confirmatory imaging modalities. Lymph nodes, lesions from direct tumor extension or along needle tracts and suture lines were excluded. Tumor histology, location and size of the primary lesion in patients with ST metastasis were categorized.

### PET/CT scanning

Patients fasted at least 4 hours before the tracer injection and received an intravenous injection of approximately 5.18 MBq/Kg (0.14 mCi/Kg) of ^18^F-FDG, with a maximum of 444 MBq (12 mCi). Blood glucose level was measured immediately prior to FDG injection and was < 200 mg in all studied cases. Patients were instructed to sit in a quiet injection room without talking during the subsequent 45–60 min of the FDG uptake phase and were allowed to breathe normally during image acquisition without specific instructions. All scans were acquired using a PET/CT scanner (Gemini; Philips Medical Systems), with an axial co-scan range of 193 cm enabling a head-to-toe (TWB) imaging in one sweep.

### CT scanning

The CT scan of the PET/CT scanner consisted of a 16 slice multi-detector helical CT. Gantry allows for a patient port of 70 cm. Parameters were as follows for 12–13 bed acquisitions (from the top of the head through the bottom of the feet): 120–140 KV and 33–100 mAs (based on body mass index), 0.5 second per CT rotation, pitch of 0.9 and 512 × 512 matrix. CT acquisition was performed before emission acquisition. CT data were used for image fusion and the generation of the CT transmission map. In all patients, the arms were placed above the patient's head for CT acquisition except in patients with head and neck cancers where the arms were placed at the patient's sides. No oral or IV contrast was used. No separate CT interpretation was performed since the CT was of suboptimal quality.

### PET scanning and image processing

Emission data were acquired for 12–13 bed positions (193 cm coverage, identical to CT protocol). Emission scans were acquired at 3 minutes per bed position. The FOV was TWB on all patients. The 3D TWB acquisition parameters consisted of a 128 × 128 matrix and 18 cm FOV with a 50% overlap. Processing consisted of the 3D Row Action Maximum Likelihood Algorithm (RAMLA) method [7].

### Image analysis

TWB PET/CT images were retrospectively evaluated on Syntegra workstation (Philips Medical Systems), by two board certified Nuclear Medicine physicians, and a log was kept to record whether cases with the suspected lesions occurred within or outside the typical LWB FOV (base of skull to upper-thigh or pelvic floor). The distribution of ST lesions was evaluated as inside or outside LWB field of view, and the lesions were grouped as in the head, upper or lower extremities. All ST lesions were evaluated semi-quantitatively using maximum standard uptake values (SUVmax); SUVmax of the ST lesions and of the liver, as reference organ, were compared. A standardized spherical region of interest of 20 cm^3 ^was placed in the mid lateral aspect of the right hepatic lobe. Computer tomographic evaluation of the ST lesions included measurement of the largest diameter and a density judgment (iso-, hyper- or hypodense compared to the surrounding tissues). Given the limited anatomical delimitation of isodense muscular lesions from the surrounding normal muscular tissue on CT, the size of these lesions was estimated on the PET study. A board-certified Oncologist assessed the impact on management and/or staging from the detection of malignancy outside the LWB FOV.

For statistical analysis, a Student t-test was used to compare the results from subcutaneous and skeletal muscle lesions. This retrospective study was approved by the Institutional Review Board and patients' informed consent was waived.

## Results

**Figure 1:**
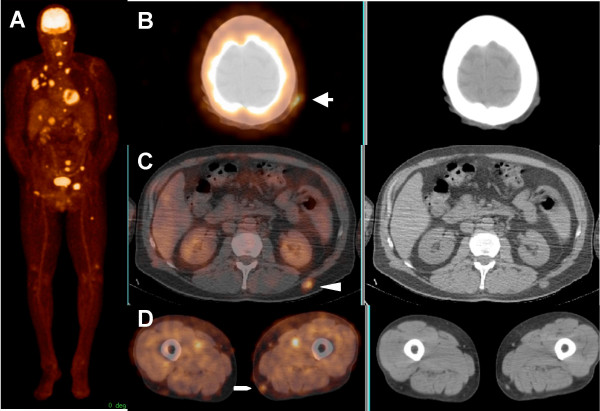
71-year-old male with history of melanoma in the right anterior chest, status post surgical resection and interleukin-2 therapy 2 weeks prior to the PET/CT scan. Maximum intensity projection (MIP) image (A) and transaxial images (B, C and D) show widespread metastatic disease including three ST lesions in the left scalp (arrow), left mid back (arrow head) and left distal thigh (pentagon).

The TWB PET/CT studies suggested the presence of ST metastasis in 11 out of 500 studied patients (2.2%). False positive findings were present in 2 of 11 patients which included one biopsy-proven actinic keratosis and one axillary skin folding which initially was misinterpreted as suspicious for skin metastasis; clinical exam of the axilla was unremarkable, and the patient has remained in complete remission by clinical exam and follow-up PET/CT. ST metastasis was confirmed in the remaining 9 patients (3 females, 6 males, age range 35–76, mean age 60). Those 9 patients had a total of 41 ST lesions. Twenty two lesions were within the LWB FOV (54%) and 19 lesions outside it (46%). Three lesions were in the head, 14 in the torso, 4 in the upper extremities and 20 in the lower extremities. Subcutaneous lesions were 19 (46%) and muscular lesions were 22 (54%). No ST metastasis was found in the following patient populations: 32 breast cancers, 45 colorectal cancers, 29 cancers of the hepatobiliary system, 50 head and neck cancers, 10 pancreatic cancers, 9 renal cell cancers, 10 cancers of the reproductive system, 12 sarcomas, 8 thyroid cancers and 91 miscellaneous cancers (unknown primary, non-specified cancer, cancer of the bone, glioblastoma etc.). Melanoma was encountered in 4 of the 9 cases (44%) and represented, with a prevalence of 4/41 (9.8%), the most frequent neoplasm with metastasis outside the LWB FOV (Figure 1). Of the 4 patients with melanoma, two had ST metastasis in close proximity to the primary lesion (scalp, thigh) which can be classified as in-transit metastasis. The remaining 2 patients had the primary lesion in left upper arm and right anterior chest. All melanoma patients presented with simultaneous widespread ST metastasis and other distant metastasis within and outside LWB. Lymphoma and lung carcinoma represented with 2 cases each and corresponded to a prevalence of 2/60 (3.3%) and 2/88 (2.3%), respectively. Although represented with only one case, esophageal cancer had a prevalence of 1/13 (7.7%) representing a higher prevalence when compared to lymphoma or lung carcinoma given the relatively small number of patients with esophageal cancer.

The distribution and clinical features of ST metastasis in the 9 patients are shown in Table 1.

**Table 1:** Distribution and clinical features of soft tissue metastasis of 9 included patients

**Patient no.**	**Gender; age (y)**	**Cancer type; initial staging**	**Location, size/grading of primary lesion**	**Inside LWB – Sq**	**Outside LWB – Sq**	**Inside LWB – Sm**	**Outside LWB – Sm**	**Months from initial Dx to Dx of STM**	**Months of survival**	**Type of follow-up**
1	M; 35	Melanoma; III	Left scalp, 4.3 mm^†^	1: lt post-auricular*	0	0	0	3	DOD, 25	B
2	M; 71	Melanoma; III	Left upper arm, size n/a	10: lt shoulder, abdomen, back, pelvis, lt thigh	2: lt elbow, lt thigh	2: rt shoulder, rt thigh	7: rt thigh, lt thigh, lt lower leg	48	DOD, 56	B
3	M; 70	Melanoma, spindle type; IV	Right anterior chest, 1.4 mm^†^	1: lt face	1: lt parietal skin	0	0	48	DOD, 53	B
4	M; 76	Melanoma; III	Right thigh, 4.8 mm^†^	0	1: rt thigh*	0	0	4	DOD, 7	B
5	F; 65	Lung cancer, squamous ; IV	Left lung, 5.5 cm	0	0	1: rt paraspinal	0	6	DOD, 7	C
6	M; 44	Lung cancer, non-small cell with squamoid features; IV	Left lung, 3.8 cm	0	1: lt thigh	2: rt shoulder, lt thigh	0	1	DOD, 9	C
7	F; 64	Lymphoma, follicular cell; IV	Widespread tumor, grade II/III	1: rt chest	0	0	0	1	NED, 29	P
8	F; 56	Lymphoma, diffuse large B-cell, anaplastic variant; II	Left groin 5.1 cm; retroperitoneum, 4.5 cm	0	1: rt elbow	1: rt thigh	3: rt thigh, lt thigh	7	n/a	C
9	M; 63	Esophagealcancer, adenocarcinoma; staging n/a	Size n/a	0	0	3: lt arm, lt pelvis	3: lt thigh, rt calf, lt calf	12	DOD, 13	C

			**Total lesion number**	**13**	**6**	**9**	**13**			
						
			**SUVmax**	5.5 ± 2.8 (1.5 – 9.6); all higher than liver uptake	8.9 ± 12.6 (1.0 – 37); 3/6 higher than liver uptake	5.8 ± 3.0 (2.2 – 10); all higher than liver uptake	3.4 ± 2.5 (0.9 – 8.4); 11/13 higher than liver uptake			
			**CT appearance**	13/13 hyperdense	6/6 hyperdense	4/9 isodense, 5/9 hypodense	8/13 isodense, 5/13 hypodense			
			**Lesion size**	8/13, < 1.5 cm; 5/13, 1.5 – 1.8 cm	4/6, < 1.5 cm; 2/6, 1.5 – 1.8 cm	1/9, < 1.5 cm; 8/9, 2.5 – 2.8 cm	7/13, < 1.5 cm; 6/13, 1.5 – 9 cm			

LWB, limited whole body; Sq, subcutaneous; Sm, skeletal muscle; rt, right; lt, left; NSCLC, non-small cell lung cancer; DOD, death of disease; NED, no evidence of disease; n/a, not available; STM, soft tissue metastasis; B, biopsy; C, clinical; P, PET/CT

* in-transit metastasis; ^† ^Breslow's depth

Seven out of the 9 patients developed ST metastasis within 12 months of diagnosis of the primary malignancy. The remaining two patients had melanoma and developed ST metastasis 4 years after the initial diagnosis. These later patients died within 5 months and 8 months, respectively, after the diagnosis of ST metastasis.

**Figure 2:**
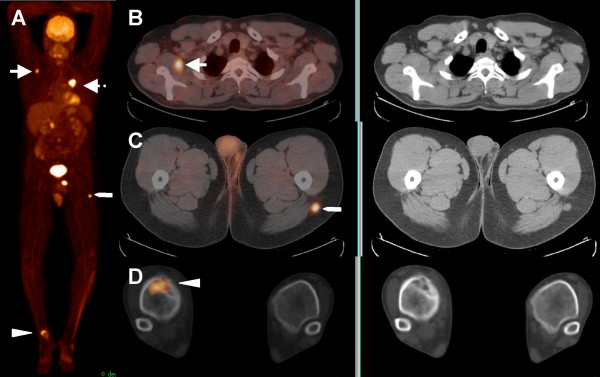
44-year-old male with history of non-small cell lung carcinoma. MIP image (A) and transaxial images (B, C and D) show the primary lesion in the left lung (dotted arrow), a single subcutaneous ST lesion in the left proximal thigh (pentagon) and a bone lesion in the right distal tibia (arrow head).

All 9 patients presented with other metastasis by the time ST metastasis was identified (see Figures 1 and 2). The presence of ST metastasis therefore neither changed the staging nor treatment decisions in any of these patients. However, the ST lesions provided a more accessible biopsy site in 4 of the 9 patients (44%). Seven out of 9 patients died of their disease. The mean duration from initial diagnosis of the disease to death was 24 months (range 7–56 months); while the mean duration from ST metastasis diagnosis to death was 7 months (range 1–22 months). The remaining 2 patients were the two lymphoma patients: one had no evidence of residual disease; and clinical data were not available for the other.

All ST metastatic lesions were FDG avid; 37/41 (90%) lesions presented with SUVmax higher than in the liver, thus, allowing easy identification of the lesions on the PET study. The remaining 4/41 (10%) had slightly less FDG uptake than in the liver and were < 1.5 cm in size; however, these lesions were identifiable as they were surrounded by normal soft tissue that did not have any significant FDG uptake. SUVmax was not statistically significant (p = 0.394) between subcutaneous lesions (mean 5.2 +/- 3.8) and skeletal muscle lesions (mean 4.3 +/- 2.9). The size of the ST metastatic lesions ranged from < 1 cm to 9 cm with 20/41 (49%) being less than 1.5 cm and the remaining 51% being between 1.5 – 9 cm. The sizes of subcutaneous lesions (mean 1.2 +/- 0.3), and skeletal muscle lesions (mean 2.2 +/- 2.2), did not differ significantly (p = 0.106); but skeletal muscle lesions tended to be larger than the subcutaneous ones. Subcutaneous lesions were easily identifiable as hyperdense lesions on CT. Skeletal muscle lesions were hypodense in 10/22 (45%) and isodense in 12/22 (55%). No hyperdense skeletal muscle lesions were noted.

## Discussion

Our study showed that 9 of 500 (1.8%) cancer patients had ST metastasis. Spencer et al. reported the prevalence of skin metastasis of any cancer type to vary between 0.75% and 9% [1]. Other studies in lung cancer patients revealed a lower and less variable cutaneous metastasis prevalence of 1.3% to 3.1% which is comparable to our findings [2,3]. The most commonly reported primary carcinomas to result in clinically recognized ST metastasis are those of the lung, kidney, and colon [8]. Our study indicated that the prevalence of ST metastasis of lung carcinoma (2.3%) was much lower as compared to that of melanoma (9.8%) which is higher than is reported in the literature [8]. Our study showed that either in-transit or distant ST metastasis was associated with other distant metastasis, and was suggestive of poor prognosis as demonstrated previously [9,10]. Of note, three of the four melanoma patients were older than 70 years. This observation might have prognostic significance as older individuals with melanoma have increased mortality as compared with younger ones [11]. However, this requires future evaluation in a larger cohort of melanoma patients. Two of 3 patients with available Breslow's depth showed lesion thickness greater than 4 mm (pT4) which is indicative of high risk neoplasms and may explain the widespread disease in these patients. Lymphoma has rarely been reported to have ST metastasis [8]. However, we found an equal number of cases with ST metastasis in the lymphoma and lung carcinoma cohorts (2 cases each). Among the lymphoma cases, one had a CD30 positive, anaplastic variant, diffuse large B-cell lymphoma potentially explaining the development of ST metastasis. The other patient had a grade III follicular lymphoma with a single subcutaneous lesion and limited lymphadenopathy. Additional research is needed to fully comprehend the prevalence and pattern of ST metastasis in lymphoma.

The most frequently reported locations for ST metastasis have been the back, chest wall, and abdomen [12]. These are the areas typically included in chest, abdomen and pelvis CT scans as well as the LWB PET/CT scans. In contrast, our study showed that ST metastasis occurred outside the typical LWB FOV in 46% of cases (19/41 lesions). Thus, previously reported prevalence and locations of ST metastasis may have been biased by the imaged FOV. ST metastasis has been reported as a common clinical presentation of occult malignancy and as an isolated metastasis in the patient with a known malignancy [8,13]. Only a small percentage of ST metastasis has been reported to occur in the presence of disseminated disease [8,14]. In contrast, our findings revealed that all patients with ST metastasis had widespread disease on PET/CT. Our study also indicated that ST metastasis can occur early during the course of the disease since 7 out of 9 patients developed ST metastasis within 12 months of diagnosis of their primary malignancy. It is likely that patients with advanced disease have been underrepresented in the literature as they neither present a diagnostic challenge nor have a curative therapy. Moreover, previous studies mostly revealed a referral bias as the reported patients had symptomatic ST lesions referred for further evaluation and management [8,11].

Although magnetic resonance imaging (MRI) is not specific for soft tissue metastasis, it has been advocated as an indispensable tool for the diagnosis and treatment planning in patients with soft tissue malignancy [15]. However, a recent study showed that F-18 FDG PET/CT has higher sensitivity than MRI in detecting skin and ST metastasis [16]. This is supportive of the increasing role of F-18 FDG PET/CT in cancer patient management [4]. Nevertheless, there are undoubtedly false positives as seen in two cases (actinic keratosis, skin folding) of the studied population that need to be taken into account. FDG uptake and resulting increased tracer activity is not limited to neoplastic tissue. Recognizing the strengths and weaknesses of PET is important for the accurate interpretation of the PET/CT images. The diagnosis of ST metastasis in our study using combined PET/CT was relatively straightforward as most lesions had significant FDG uptake higher than that of the liver which is a widely accepted reference organ to distinguish benign from malignant lesions. Most PET facilities recommend at least 4 hours of fasting before the tracer injection as a standard. A longer fasting time may increase the detection of ST lesions; however, the standard protocol of at least 4 hours fasting was followed in this retrospective study. PET/CT protocol in cancer staging usually comprises a low dose, non-enhanced CT protocol [17,18], which is sufficient for attenuation correction and anatomical information while keeping the radiation exposure to a minimum. Given the low-dose and non-contrast enhanced protocol, the CT portion of the study helped localize the lesions and increase the diagnostic confidence as ST metastatic lesions can appear hyperdense or hypodense as compared to the surrounding soft tissue.

ST metastasis can be present in many muscular and subcutaneous sites across the body with a ratio higher than 1.5:1 [8]. In our study, the ratio was 1.2:1, suggesting that subcutaneous ST metastasis may have been under-reported in the literature. One explanation for this may be that subcutaneous lesions tended to be smaller than muscular ones, although our findings did not reveal a statistically significant difference in these lesions' size (p = 0.106). Another potential reason is that 5/19 (26%) of the subcutaneous lesions in our study were 1 cm or less in size which may represent a diagnostic limitation for diagnostic CT and MRI scans.

Certainly, the prognosis in the presence of ST metastasis should be considered when weighing the merits of the findings. Seven out of nine studied patients died of their disease within 1–22 months after ST metastasis was diagnosed. This correlates with the reported median survival ranging from less than 5 months to no greater than 19 months after the diagnosis of ST metastasis [8].

We acknowledge the limitations of our retrospective study. We also realize that at many institutions, a TWB imaging is frequently performed in melanoma patients and probably would have detected the in-transit metastasis in two of the four melanoma patients (scalp, thigh). We intended to evaluate a cohort of 500 consecutive cancer patients with TWB PET/CT imaging and tried to delineate the extent of ST metastasis in this population. PET/CT is a relatively new technology that has already been shown to benefit the management of a number of cancers [18,19]. A recent literature-based evidence review reported an average of 15% improvement in staging and restaging accuracies of PET/CT over PET or CT alone in different cancers [4]. The superior ability of F-18 FDG-PET/CT in the detection of metastatic disease can help provide an easily accessible biopsy site and avoid unnecessary invasive diagnostic procedures as it was the case in 4 of our 9 patients (44%). This can result in less invasive procedures performed, decreasing morbidity and cost.

LWB PET/CT scanning is typically performed from the skull base to the pelvic floor [5,6] because most FDG avid lesions are expected to be within this field of view excepting cerebral metastasis which can be found in at least 20% of cancer patients during their life-time [20]. However, the sensitivity of F-18 FDG PET is suboptimal in detecting brain metastases due to the intense physiologic background uptake in the brain and the hypometabolic nature of some brain metastases [21]. Because of the higher sensitivity and specificity of contrast-enhanced MRI for cerebral metastasis [21], the use of F-18 FDG PET/CT to diagnose brain metastasis has become less desirable. Despite multiple reports in the literature, the prevalence of distant metastasis to the extremities is rare [1,2,4,8,12,22,23]. This is most likely why the extremities are usually not included in the field of view unless there is a clinical suspicion for cancer in the extremities. In our study, LWB scanning would have under-diagnosed all lesions outside LWB FOV. We found that 19/41 (46%) of ST metastatic lesions were detected outside the LWB FOV, i.e. could only be detected by TWB scanning.

A decision whether a TWB scanning should be used in cancer patients depends on the overall prevalence of distant metastasis outside the LWB FOV which is not limited to ST metastasis alone. The added value of TWB scan over LWB scan is beyond the scope of this manuscript. However, analyzing the same patient cohort of the current study, we found that distant metastasis occurred outside of LWB FOV in 28/500 (5.6%) patients [24]. The detection of such lesions had direct patient management in about 50% of the patients because of upstaging (unpublished data). The tumors with the highest prevalence (> 10%) of distant metastasis outside the LWB were melanoma and lung cancer. Therefore, TWB imaging for malignant melanoma and lung cancer would be a reasonable option and most beneficial for these two malignancies. However, metastasis outside of LWB in other cancers has the potential pitfall of a low overall prevalence of distant metastasis outside the LWB FOV. Of note, TWB imaging requires additional several minutes of image acquisition which can be uncomfortable for the patients and results in decreased scanning throughput; however, the time required for TWB image acquisition will continue to decrease with advancements in both hardware and software technology in newer PET/CT scanner designs that would allow increased scanning throughput without compromising imaging accuracy in an economical sense. For example, the recently installed PET/CT scanner at our institution is capable of acquiring a TWB scan in a patient with a normal body mass index in less than 18 minutes.

## Conclusion

The detection of ST metastasis may have prognostic implications, provide more accessible biopsy sites and help avoid invasive procedures. A LWB scanning may underestimate the true extent of ST metastasis since a significant percentage of ST metastasis (46%) occurred outside the typical LWB FOV.

## Competing interests

The author(s) declare that they have no competing interests.

## Authors' contributions

All authors read and approved the final manuscript. NCN collected clinical data, reviewed the PET/CT scans and carried out measurements of SUVmax and lesion size and wrote the manuscript. BTC helped review clinical data, assessed the impact of soft tissue metastasis on staging and management and assisted in writing the manuscript. MMO initiated, design and supervise the study, review the PET/CT scans and assisted in writing the manuscript.

## Pre-publication history

The pre-publication history for this paper can be accessed here:


